# The influence of age on insecticide susceptibility of *Anopheles arabiensis* during dry and rainy seasons in rice irrigation schemes of Northern Tanzania

**DOI:** 10.1186/s12936-017-2022-6

**Published:** 2017-09-11

**Authors:** Saada Mbepera, Gamba Nkwengulila, Rose Peter, Emmanuel A. Mausa, Aneth M. Mahande, Maureen Coetzee, Eliningaya J. Kweka

**Affiliations:** 1 Department of Zoology and Wildlife Conservation, College of Natural and Applied Sciences, University of Dar-es-salaam, P.O.Box 35165, Dar-es-salaam, Tanzania; 2 Public Health Strategic Partnerships Associate, Arysta Life Science, 12 Denys Road, River Club, 2191 South Africa; 3 National Plant Genetic Resource Centre, Tropical Pesticides Research Institute, P.O. Box 3024, Arusha, Tanzania; 4 Division of Livestock and Human Diseases Vector Control, Tropical Pesticides Research Institute, Mabogini Field Station, Moshi, Tanzania; 5 Wits Research Institute for Malaria, School of Pathology, Faculty of Health Sciences, University of the Witwatersrand, Johannesburg, South Africa; 6 Centre for Emerging, Zoonotic & Parasitic Diseases, National Institute for Communicable Diseases, Johannesburg, South Africa; 7 Division of Livestock and Human Diseases Vector Control, Tropical Pesticides Research Institute, P.O.Box 3024, Arusha, Tanzania; 8 Department of Medical Parasitology and Entomology, School of Medicine, Catholic University of Health and Allied Sciences, P.O. Box 1464, Mwanza, Tanzania

**Keywords:** *Anopheles arabiensis*, Age, Seasons, Insecticides, Resistance, Pyrethr

## Abstract

**Background:**

Insecticide resistance is the major emerging challenge facing the malaria vector control programmes in Tanzania. Proper monitoring and detection is of paramount importance guiding the vector control programmes. This paper presents the effect of mosquito aging on insecticide resistance status in *Anopheles arabiensis* populations in dry and rainy seasons in northern Tanzania.

**Methods:**

*Anopheles gambiae s.l.* larvae were sampled from rice fields in both dry and rainy seasons and reared in the insectary to adults. The emerged females in batches of 2, 3, 5, and 10 days old were exposed to six insecticides (deltamethrin, permethrin, lambda-cyhalothrin, DDT, bendiocarb and pirimiphos-methyl) to see the effects of age on insecticide resistance. Mosquitoes were exposed to insecticides using WHO standard susceptibility test kits. Knockdown was recorded during the 1-h exposure, while mortality and resistance ratio were recorded 24 h later. Mosquito specimens were identified to species level using the polymerase chain reaction (PCR) method.

**Results:**

Among the 326 specimens processed by PCR, 323 (99.1%) were identified as *Anopheles arabiensis*. There was reduced mortality (ranging from 61 to 97.7%) when adults reared from larvae were exposed to all pyrethroids and bendiocarb in both dry and rainy seasons, while they were fully susceptible to DDT and pirimiphos-methyl. There was a significant increase in mortality rate with increase in mosquito’s age in both dry and rainy seasons following exposure to pyrethroids (DF = 1, *P* < 0.05). Mosquitoes showed significantly higher mortality rates in the rainy season than in the dry season after being exposed to pyrethroids (DF = 1, *P* < 0.05). Higher mortality rates (94.0–99.8%) were observed in all ages and seasons when mosquitoes were exposed to bendiocarb compared with pyrethroids. Pirimiphos-methyl was only tested in the rainy season so no comparison with dry season mosquitoes could be made.

**Conclusions:**

Results showed that *An. arabiensis* were resistant to pyrethroids in both seasons and that the young age groups exhibited higher levels of resistance compared with the older age groups. Mosquitoes were full susceptible to DDT and pirimiphos-methyl irrespective of the season and age.

## Background

Malaria vector control mostly relies on indoor residual house spraying (IRS) and long-lasting insecticide-treated bed nets (LLINs) [1] and has accounted for the dramatic decline in malaria transmission over the past decade. Malaria control and subsequently elimination are the priority of sub-Saharan African countries through national malaria control programmes and other donors [2]. In Tanzania, wide coverage and use of LLINS began in 2005 and has been a sustainable exercise to date with aid from different funders [3–5].

Insecticide resistance in Tanzania has been reported widely in the major malaria vectors *Anopheles gambiae s.s., Anopheles arabiensis* and *Anopheles funestus* [6–8] causing concern for the vector control programmes. Only four classes of insecticides are approved for use in vector control: pyrethroids (the only class used for LLIN treatment), organophosphates, carbamates and organochlorides [9]. Pyrethroids is the only class of insecticides widely used in sub-Saharan Africa for both LLINs and IRS and the rapidly spreading resistance to these insecticides is a challenge and major drawback to the gains achieved in malaria control to date [10–12]. Organophosphates and carbamates have been shown to have higher efficacy than pyrethroids for IRS [13–16]. However, in some areas of Tanzania there is evidence that mosquitoes populations are resistant to carbamate [14, 17]. The need for new insecticide classes is of paramount importance for handling and managing vector resistance [18].

In Tanzania, insecticide resistance is linked with agricultural activities [19–21], use of LLINs and IRS programmes with wide coverage [14, 22]. The expanding irrigation programmes for food security and cash crops cultivation such as cotton have increased the use of pesticides which subsequently is associated with resistance build-up in mosquitoes [7, 23]. The observed resistance is closely related to the distribution of the *An. gambiae* species complex and different mechanisms associated with insecticide tolerance [6–8, 12]. Both *kdr* mutations, L1014S and L1014F, have been found to occur in both *An. arabiensis* and *An. gambiae s.s.* in different populations in different parts of Tanzania [6]. The L1014F mutation reduces sensitivity to both pyrethroids and DDT [24].

It is anticipated that, in areas with intense insecticide resistance, the survivorship of vector mosquitoes will be higher despite the use of LLINs and IRS [25]. The association between insect fitness and insecticide tolerance may decrease or increase the vectorial capacity of mosquitoes [25]. In general, mosquitoes lose their insecticide tolerance as age increases [26]. The effectiveness of LLINs and IRS is due to their efficiency in reducing daily survivorship of vector mosquitoes and for LLINs, producing a physical barrier between the mosquito and its host. If insecticide tolerance decreases with aging, then the intervention tools will continue offering the maximum protection. Furthermore, there is no evidence on the effect of seasonality on the susceptibility status of mosquito populations of different ages in Tanzania.

The objectives of this study, therefore, were to determine aging and seasonality effects on insecticide resistance status of wild populations of *Anopheles gambiae s.l.* in northern Tanzania.

## Methods

### Study area

This study was conducted at the Lower Moshi rice irrigation scheme in Mabogini village situated at the foot of mount Kilimanjaro (37°20′E, 03°21′S: 750 m above sea level). The area is characterized by a tropical climate with heavy rains from March to May (18–27 °C) and a short rainy season during October to December (17–28.5 °C). A hot dry season occurs from January to February (17–30 °C) while the cold dry season is from June to September (13.7–24.8 °C) [27].

### Collection and rearing of wild *An. gambiae s.l.* larvae

Larvae of different life stages (L1–L4) of *An. gambiae s.l.* were collected within rice farms at Mabogini during the dry season (July–September 2013) and the rainy season (March–May 2014). The Lower Moshi rice schemes are irrigated throughout the year and ensure continuous breeding sites for mosquitoes. In the laboratory, the collected larvae were re-distributed evenly in development trays with habitat water, and provided with Nestle Cerelac mixed with fish powder (ratio 3:1) once a day. Larvae were reared to adults under insectary conditions of 27 ± 2 °C and 78 ± 2% relative humidity at TPRI, Mabogini field station with photophase of 12L:12D.

### Insecticide susceptibility tests

The Kisumu susceptible laboratory strain was used as a positive control for all exposures to ensure that the test papers were effective. To determine the influence of age on insecticide resistance status, non blood-fed emergent adult females were held for 2, 3, 5, and 10 days after emergence before exposure to insecticides. The age structure used was based on previous studies used that age as the determinant of resistance limit [28–30]. Standard WHO susceptibility test kits were used. Six insecticides were tested: deltamethrin (0.05%), permethrin (0.75%), lambda-cyhalothrin (0.05%), bendiocarb (0.1%), DDT (4.0%) and pirimiphos-methyl (0.025%). Only female *An. gambiae s.l.* were used for the susceptibility tests according to WHO criteria [9]. Each complete bioassay was performed with six batches of 20 unfed females of the same age. Four batches were exposed to treated filter papers, and two batches exposed to oil treated filter papers (negative control). The numbers of mosquitoes knocked down after contact with insecticides were recorded at the recommended intervals [9]. The exposure period was 1 h and mortality was determined 24 h post-exposure. After being exposed to insecticide for 1 h, mosquitoes were provided with a 10% sugar solution for 24 h before the mortality score.

### Species identification

All specimens were identified to species using the standard DNA PCR method for the *An. gambiae* complex [28]. DNA samples were extracted from a single leg or wing and processed according to the protocol of Scot et al. [31].

### Data analysis

Data were entered in MS-excel and transferred to PASW Statistics version 18.0 for Windows (SPSS Inc., Chicago, IL) for analysis. Regression probit analysis was deployed to calculate the KDT_50_ and KDT_95_ of the population by season, insecticide type and mosquito age. Percent mortalities were used to determine the insecticide susceptibility/resistance status of the population during both dry and rainy seasons using one way Anova with Tukeys HSD test to separate the significance difference between the means. The field-collected mosquitoes KDT_50_ was compared with that of the *Anopheles gambiae*, Kisumu susceptible strain by estimates of KDT_50_ ratios (Resistance Ratio). The significant levels were considered at 5% and less.

## Results

### Species composition in study site

**Fig. 1:**
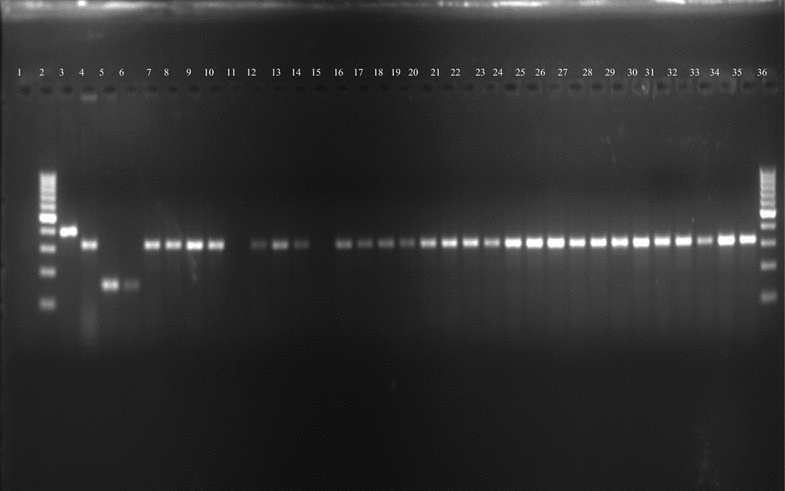
Species identification for wild emerged adults of *Anopheles gambiae s.l.* (*Lane 1* negative control, *Lanes 2* and *36* DNA ladder, *Lane 3 Anopheles gambiae* control, *Lane 4 Anopheles arabiensis* control, *Lane 5 Anopheles quadriannulatus* control, *Lane 6 Anopheles merus* control, *Lanes 7*–*35* wild mosquitoes—all *An. arabiensis*)

A total of 326 specimens of *An. gambiae s.l.* were processed by PCR and 323 (99.1%) were identified as *An. arabiensis* (Fig. 1). Three specimens did not amplify.

### Insecticide susceptibility for wild and laboratory Kisumu strain populations

The knock down times for 50 and 95% (KDT_50_ and KDT_95_) of the wild and laboratory colony population varied with insecticide type. For DDT, the mean KDT_50_ and mean KDT_95_ were higher in the rainy season than in the dry season for all ages (Table 1). For lambda-cyhalothrin, the rainy season had higher KDT_50_ and KDT_95_ at the age of 2, 3 and 5 days while for day 10, KDT_50_ and KDT_95_ was higher in the dry season than the rainy season (Table 2). For Deltamethrin, at the age of 2 and 5 days KDT_50_ and KDT_95_ was higher in the dry season than in the rainy season while for mosquitoes of 3 and 10 days old, KDT_50_ and KDT_95_ was higher in the rainy season than in the dry season (Table 3). For Permethrin, at the age of 2, 3 and 5 days old, KDT_50_ and KDT_95_ was higher in the dry season than the rainy season and alternated at the age of 10 days, with higher KDT_50_ in the rainy season and higher KDT_95_ in the dry season (Table 4). For Bendiocarb, KDT_50_ and KDT_95_ for the age of 2,3, and 5 days old was higher in the dry season than the rainy season while at 10 days old the KDT_50_ and KDT_95_ was higher in the rainy season than the dry season (Table 5). For pirimiphos-methyl, KDT_50_ and KDT_95_ for the age of 2, 3 5 and 10 days in the rainy season increased with aging (Table 6). The Kisumu *An. gambiae* susceptible colony was used as controls for KDT_50_ and KDT_95_ for each season and each insecticide (Tables 1, 2, 3, 3, 5 and 6).

**Table 1:** Knock-down time and mortality rates of *Anopheles arabiensis* exposed to DDT for a period of 60 min in dry and rainy seasons

Age (days)	Season	Number tested	24 h mortality	95% CI	KDT_50_	95% CI	KDT_95_	95% CI	KDT_50_ ratio (RR)
2	Dry	800	100	–	24.5	23.8–25.3	46.1	44.4–47.9	1.3
	Rain	800	100	–	26.8	22.7–31.6	52.8	44.2–64.7	1.7
3	Dry	800	100	–	27.2	26.2–28.2	51.2	49.1–53.4	1.5
	Rain	800	100	–	28.4	25.1–32.1	55.8	48.4–66.4	1.8
5	Dry	800	100	–	22.58	21.8–23.4	42.5	40.8–44.4	1.2
	Rain	800	100	–	26.7	23.5–30.4	52.7	45.6–62.5	1.7
10	Dry	800	100	–	25.3	24.5–26.2	47.7	47.8–49.7	1.4
	Rain	800	100	–	25.4	22.4–28.9	50.1	43.3–59.5	1.6
Control^a^	Dry	800	100	–	18.2	–	23.7	–	–
	Rain	800	100	–	15.9	–	21.3	–	–

^a^Control was *Anopheles gambiae* Kisumu strain aged 2 days old

**Table 2:** Knock-down time and mortality rates of *Anopheles arabiensis* exposed to Lambda-cyhalothrin for a period of 60 min in dry and rainy seasons

Age (days)	Season	Number tested	24 h mortality	95% CI	KDT_50_	95% CI	KDT_95_	95% CI	KDT_50_ ratio (RR)
2	Dry	800	40.4	36.1–44.7	51.36	49.2–53.7	126.3	118.6–135.1	3.4
	Rain	800	76.0	73.7–78.3	18.82	18.1–19.6	46.07	44.8–49.2	1.4
3	Dry	800	43.1	28.8–57.4	57.10	54.6–59.7	140.4	131.5–150.7	3.8
	Rain	800	81.0	78.7–83.3	33.35	32.2–34.3	83.1	79.4–87.1	2.4
5	Dry	800	79.6	57.0–102.2	50.33	48.1–52.6	123.7	116.2–132.3	3.3
	Rain	800	91.8	88.9–94.6	26.39	25.4–27.4	53.7	51.5–56.2	1.9
10	Dry	800	81.7	66.7–96.6	23.9	22.9–25.0	58.9	55.7–62.5	1.6
	Rain	800	97.8	96.5–99.1	34.14	32.9–35.3	84.9	81.2–89.1	2.5
Control^a^	Dry	800	100	–	15.1	–	27.6	–	–
	Rain	800	100	–	13.7	–	27.3	–	–

^a^Control was *Anopheles gambiae* Kisumu strain aged 2 days old

**Table 3:** Knock-down time and mortality rates of *Anopheles arabiensis* exposed to Deltamethrin for a period of 60 min in dry and rainy seasons

Age (days)	Season	Number tested	24 h mortality	95% CI	KDT_50_	95% CI	KDT_95_	95% CI	KDT_50_ ratio (RR)
2	Dry	800	44.5	39.9–49.2	36.6	34.3–37.9	101.2	94.6–37.9	2.9
	Rain	800	48.9	42.6–55.3	33.5	32.5–34.6	72.9	70.3–75.8	2.4
3	Dry	800	50.14	45.9–54.4	15.9	14.9–16.8	44.6	41.7–47.8	1.3
	Rain	800	52.4	46.4–58.5	30.9	30.0–31.9	67.3	64.9–69.9	2.2
5	Dry	800	49.9	44.4–55.4	32.7	31.8–33.7	71.1	68.6–73.8	2.6
	Rain	800	60.8	55.0–66.6	21.3	20.13–22.5	59.8	55.9–64.2	1.5
10	Dry	800	60.5	54.7–66.3	25.1	24.3–25.8	54.5	52.5–56.6	2.0
	Rain	800	71.9	67.0–76.9	27.9	26.8–29.0	78.3	73.9–83.4	2.0
Control^a^	Dry	800	100	–	12.5	–	19.8	–	–
	Rain	800	100	–	13.9	–	21.2	–	–

^a^Control was *Anopheles gambiae* Kisumu strain aged 2 days old

**Table 4:** Knock-down time and mortality rates of *Anopheles arabiensis* exposed to Permethrin for a period of 60 min in dry and rainy seasons

Age (days)	Season	Number tested	24 h mortality	95% CI	KDT_50_	95% CI	KDT_95_	95% CI	KDT_50_ ratio (RR)
2	Dry	800	30.8	25.7–35.8	51.7	49.5–54.1	144.9	135.9–155.3	4.1
	Rain	800	55.3	52.9–57.6	48.2	46.6–49.9	122	116.4–128.5	3.8
3	Dry	800	43.7	38.7–48.8	37.7	36.1–39.3	105.5	99.6–112.3	3.0
	Rain	800	88.8	86.3–91.2	25.5	24.6–26.4	64.5	61.7–67.6	2.0
5	Dry	800	43.8	38.8–48.8	37.3	35.8–38.9	104.6	98.6–111.3	3.0
	Rain	800	93.0	91.6–94.4	16.0	15.4–16.6	40.6	38.8–42.5	1.3
10	Dry	800	64.8	59.1–70.4	22.2	21.2–33.3	62.36	58.8–66.3	1.8
	Rain	800	97.5	95.6–99.4	24.5	23.7–25.3	62.0	59.4–64.9	1.9
Control^a^	Dry	800	100	–	12.5	–	25.1	–	–
	Rain	800	100	–	12.6	–	24.9	–	–

^a^Control was *Anopheles gambiae* Kisumu strain aged 2 days old

**Table 5:** Knock-down time and mortality rates of *Anopheles arabiensis* exposed to Bendiocarb for a period of 60 min in dry and rainy seasons

Age (days)	Season	Number tested	24 h mortality	95% CI	KDT_50_	95% CI	KDT_95_	95% CI	KDT_50_ ratio (RR)
2	Dry	800	94.0	91.7–96.3	59.3	57.5–61.2	125.2	119.7–131.3	4.5
	Rain	800	95.7	94.0–97.5	25.7	24.5–26.9	57.4	54.1–61.0	2.0
3	Dry	800	95.3	92.9–97.6	47.5	46.2–48.9	100.3	96.3–104.7	3.6
	Rain	800	96.8	95.4–98.1	25.1	24.1–26.2	56.1	53.1–59.4	1.9
5	Dry	800	98.0	97.1–98.9	44.1	42.7–45.6	93.1	89.1–97.6	3.3
	Rain	800	98.8	97.7–99.8	26.8	24.3–29.4	59.8	54.1–66.3	2.1
10	Dry	800	99.3	98.4–100.1	28.9	28.1–29.9	61.2	58.8–63.8	2.2
	Rain	800	99.8	99.1–100.4	39.2	37.7–40.7	87.5	82.8–92.8	3.0
Control^a^	Dry	800	100	–	13.3	–	27.1	–	–
	Rain	800	100	–	12.9	–	27.3	–	–

^a^Control was *Anopheles gambiae* Kisumu strain aged 2 days old

**Table 6:** Knock-down time and mortality rates of *Anopheles arabiensis* exposed to Pirimiphos-methyl for a period of 60 min in dry and rainy seasons

Age (days)	Season	Number tested	24 h mortality	95% CI	KDT_50_	95% CI	KDT_95_	95% CI	KDT_50_ ratio (RR)
2	Dry	–	–	–	–	–	–	–	–
	Rain	800	100	0	23.4	22.2–24.7	40.8	38.4–43.5	1.7
3	Dry	–	–	–	–	–	–	–	–
	Rain	800	100	0	23.3	22.1–24.6	40.6	38.2–43.3	1.7
5	Dry	–	–	–	–	–	–	–	–
	Rain	800	100	0	31.6	30.0–33.1	54.9	51.9-58.4	2.3
10	Dry	–	–	–	–	–	–	–	–
	Rain	800	100	0	36.2	34.6–37.9	63.0	59.7–66.9	2.6
Control^a^	Dry	–	–	–	–	–	–	–	–
	Rain	800	100	–	13.8	–	25.1	–	–

^a^Control was *Anopheles gambiae* aged 2 days old

The KDT_50_ of the wild populations were compared with those for the Kisumu strain (2 days old) to obtain the resistance ratio (RR) for each age group and each insecticide. The results are presented in Tables 1, 2, 3, 3, 5 and 6.

### Age structure, seasonality and resistance

**Fig. 2:**
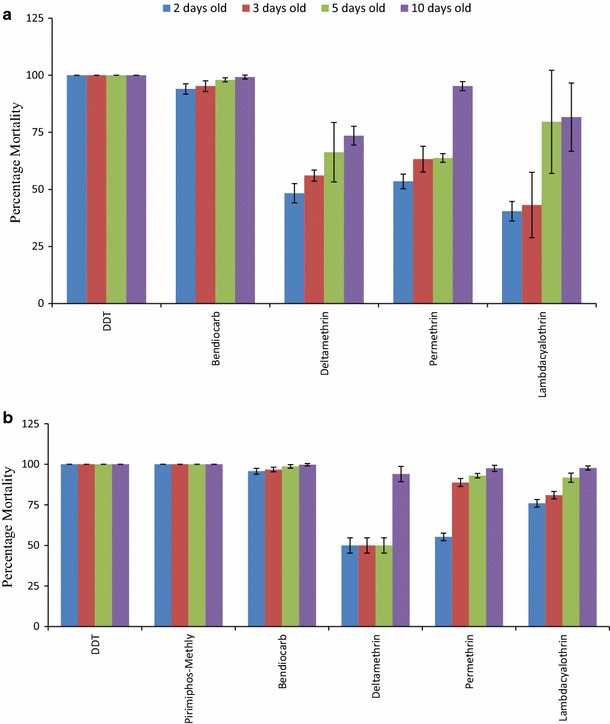
*Anopheles arabiensis* mortality 24 h after exposure to different insecticides by age in **a** dry season and **b** rainy season

There was significant difference in mortality depending on the age of adults tested and insecticide used. Mortality was age dependant for all pyrethroids with more young mosquitoes surviving than the older age groups in both dry and rainy seasons (Fig. 2). There was significant difference in mortality between the four age structures tested with pyrethroids in both seasons (DF = 2, *P* < 0.001) but no difference between the two seasons (DF = 1, P > 0.05) (Tables 2, 3 and 4). For bendiocarb, mortality ranged between 94 and 99.7% in both seasons, and there was no significance difference in mortality between the age groups and the season (DF = 1, *P* > 0.05). Full susceptibility (100% mortality) was observed when all age groups of *An. arabiensis* were exposed to DDT in both seasons and pirimiphos-methyl in the rainy season (Fig. 2).

## Discussion

The findings of this study have shown that the wild population of *An. arabiensis* from Mabogini exhibits different levels of mortality to the three pyrethroids tested depending on the age of the mosquitoes. These results are similar to previous studies that found mortality to be age dependant but did not include seasonality as a variable [26, 28, 32]. The higher resistance of the young mosquitoes to pyrethroids could be due to their strength and physiological activeness compared to older ones [28, 32]. This is because mosquitoes may lose energy due to aging, which is also needed for resistance mechanisms, thus mosquitoes reduce their ability to adapt to environmental stress [32]. Similar observations were reported from Côte d’lvoire with increased mortality associated with mosquito age following exposure to deltamethrin and permethrin [28, 32]. The increase of mortality to pyrethroids with increase in mosquito age has been found in other malaria vector species, including *Anopheles sinensis* [33] and *An. funestus* [34, 35].

Mosquitoes exhibited higher resistance to pyrethroids in the dry season than in the rainy season but they were not statistically different in some age groups, in deltamethrin statistical significant variation between rainy and dry seasons was observed in 10 days old mosquitoes (Table 3), for lambdacyalothrin significant difference between rainy and dry seasons was only in age of 2 and 3 days old (Table 2). In permethrin all ages (2, 3, 5 and 10 days old) had significant variation between seasons (Table 4). This might possibly be attributed to high concentrations of insecticides in the rice fields where larvae were collected, compared to the rainy season when insecticide concentration would be diluted by the rains, though further investigation is needed to explore this. The higher mortality rates in adult *An. arabiensis* exposed to bendiocarb and DDT in both seasons might be due to restricted use of these insecticides [20], and likewise for pirimiphos-methyl which was only tested during rainy season. Similar results were observed for three years in wild populations of *An. funestus* and *An. arabiensis* in Malawi between 2011 and 2015 [36].

The seasonal variations in the mortality rates on pyrethroids (deltamethrin, permethrin and lambda-cyhalothrin) might possibly be due to the fluctuation of environmental factors such as temperature and humidity. In previous studies it was found that temperature and humidity have a significant role in influencing the susceptibility of mosquitoes to different insecticides [9]. The observed elevated resistance to Bendiocarb was suggested to be due to increased metabolic activities of the enzymes [15, 16].

Susceptibility of *An. arabiensis* to pirimiphos-methyl and DDT is probably due to the limited use of these insecticides for malaria control and hence mosquitoes are not exposed to them. Susceptibility of *An. gambiae s.l.* to pirimiphos-methyl was also observed in Benin [15] and in *An. funestus* in Zambia and Zimbabwe [37]. But DDT susceptibility can also be due to the underlying mechanism for resistance in the study area which is detoxification by the enzyme Glutathione-s-transferases (GST) which was reported previously [8].

## Conclusions

The current study has confirmed that *An. arabiensis* is more resistant to pyrethroids than other insecticides in both the dry and rainy seasons, and the younger age groups exhibited higher levels of resistance than older age groups. Mosquitoes were fully susceptible to DDT and Pirimiphos-methyl in irrespective of the season and age. These results should be taken into account by malaria vector control stakeholders when considering the selection of appropriate insecticides for resistance management strategies.

## Abbreviations

CI: confidence interval
DDT: dichloro-diphenyl-trichloroethane
GSTs: glutathione- s-transferases
IRS: indoor residual spray
KDT_50_: time taken for 50% of the sample to be knocked down
KDT_95_: time taken for 95% of the sample to be knocked down
LLINs: long lasting insecticide-treated bed nets
PCR: polymerase chain reaction
WHO: World Health Organization
